# Overexpression of *SlGRAS7* Affects Multiple Behaviors Leading to Confer Abiotic Stresses Tolerance and Impacts Gibberellin and Auxin Signaling in Tomato

**DOI:** 10.1155/2019/4051981

**Published:** 2019-07-01

**Authors:** Sidra Habib, Muhammad Waseem, Ning Li, Lu Yang, Zhengguo Li

**Affiliations:** School of Life Science, Chongqing University, Chongqing 400044, China

## Abstract

Abiotic stresses remain the key environmental issues that reduce plant development and therefore affect crop production. Transcription factors, such as the GRAS family, are involved in various functions of abiotic stresses and plant growth. The GRAS family of tomato (*Solanum lycopersicum*), *SlGRAS7*, is described in this study. We produced overexpressing *SlGARS7* plants to learn more about the GRAS transcription factors. Plants overexpressing *SlGARS7* (*SlGRAS7*-OE) showed multiple phenotypes related to many behaviors, including plant height, root and shoot length, and flowering time. We observed that many genes in the *SlGRAS7*-OE seedlings that are associated with auxin and gibberellin (GA) are downregulated and have altered sensitivity to GA_3_/IAA. *SlGRAS7* was upregulated during abiotic stresses following treatment with sodium chloride (NaCl) and D-mannitol in the wild-type (WT) tomato. Tomato plants overexpressing *SlGRAS7* showed more resistance to drought and salt stress comparison with WT. Our study of *SlGRAS7* in tomato demonstrates how GRAS showed an integrative role, improving resistance to abiotic stresses and enhancing gibberellin/auxin signaling through reproductive as well as vegetative processes.

## 1. Introduction

The relationship between molecular developments and environmental clues is measured by the important modifications in gene regulatory networks (GRNs), which play a dynamic part in the manipulability of development and growth in plants [1–3]. Transcriptional regulation, where the transcription factors (TFs) control a succession of target genes in a spatiotemporally particular sequence, is an important member of GRNs [2, 4]. Transcription factors from numerous plant varieties show significant functions in stress responses [5], including bZIP, MYC/MYB, ERF, NAC, WRKY, and Dof. The GRAS gene family (named after GAI, RGA, and SCR) is induced through various abiotic stresses [6, 7]. Transcription factors, such as the GRAS proteins, which are involved in plant growth and pathways of signal transduction, are involved in lateral shoot development [8], phytochrome signaling [9], gametogenesis [10], auxin signaling [11, 12], and gibberellin signaling and biosynthesis [13, 14]. GRAS proteins are categorized into 13 subfamilies, containing HAM, AtSCR, AtSCL3, AtSCL4/7, AtSCL9, AtSCL28, AtSHR, AtLAS, AtPAT1, Os4, Os19, DELLA, and Pt20 in *Arabidopsis*, rice, and Populus according to the phylogenetic tree and amino acid sequence alignment [15]. Only a few members of GRAS proteins have been functionally known and involve in signal transduction pathways and plant development. Normally, C-terminus of GRAS proteins has conservative domains, containing PFYRE SAW, VHIID, leucine heptad repeat I (LHR I), and leucine heptad repeat II (LHR II) [16–18]. However, GRAS proteins differ in sequence and length in their N-terminus, which is likely a key element to the functional specificity of each protein [19]. In addition, some experimental evidence confirms that GRAS proteins play vital roles when plants are subject to biotic or abiotic stress. A GRAS transcription factor obtained from *Vitis amurensis* was used to create transgenic *Arabidopsis*, and the overexpression of *VaPAT1* leads to drought tolerance, high salinity, and cold stress [20]. To identify the functions of other GRAS genes in crops, it will help to reveal the pathways that regulate resistance and tolerance to stress and help in the breeding of tolerant species as has been done in tomato plants. Abnormal expression of the *PeSCL7* gene enhanced resistance under drought and salt stresses in *Arabidopsis* [21] and silenced *SlGRAS6* plants expressed enhanced tolerance to disease in tomato [22]. In rice, the overexpression of *OsGRAS23* increased oxidative and drought stress resistance [23]. In rice and barley, overexpressing miR171 disturbs floral meristem determinacy and phase transitions [24, 25]. By inhibition of miR156-targeted SPL proteins, the miR171-GRAS component controls trichome distribution and flowering time [26]. This component is also critical for stimulating GA-DELLA signaling in the organization of leaf development in the light and regulation of chlorophyll biosynthesis [27]. In addition, the role of miR171 has been widely studied under several stresses in different varieties, such as maize, barley, Arabidopsis, and potato [28–31]. Thus, many studies showed that GRAS proteins play many significant functions in the tolerance of abiotic or biotic stress.

The actions of the GRAS proteins from DELLA and SCARECROW-like (SCL), gibberellin (GA), and auxin are intimately related to abiotic stress responses and growth processes in plants. Two types of GRAS interact as a complex, AtSCL3 function as a coordinator of SHR-SCR and DELLAs to aid in the cell enlargement of the root endodermis to mediate gibberellin stimulation [32, 33]. *AtRGA* [13], *AtRGL1-3* [14], and *AtGAI* [34] are DELLA mutants and have been shown to be insensitive to GA. This revealed that increased gibberellin content reduces drought resistance; however, decreased gibberellin content enhances drought resistance [35]. Through a common pathway, the primary participants of gibberellin signaling are DELLA proteins that therefore constrain growth and increase stress resistance [36]. CsSCL1 (*Castanea sativa* SCL1) in chestnut and PrSCL1 (*Pinus radiata* SCL1) in pine control adventitious root development through the regulation of auxin signaling [12]. In *Arabidopsis*, LAX3 and AUX1 are auxin influx carriers, which, combined with the SHR-SCR complex, correlate with lateral and primary root formation [37]. Auxin coordinates the expression of various genes that directly or indirectly respond to stress, and various genes that respond to auxin are controlled through abiotic stresses [38]. In addition, by inducing ROS detoxification enzymes directly or indirectly by affecting the stability of DELLA proteins, it showed that auxin can control ROS homeostasis, which revealed that GA and auxin could coordinate with one another in stress environments [39, 40].

Tomato (*Solanum lycopersicum*) is an important crop because of its great nutritive and commercial value and also a good model plant for fleshy fruit development. However, most GRAS proteins have not been functionally studied in tomato till now. It has been showed that the GRAS family has 53 members in tomato [41]. Overexpression of *SlGRAS24* and *SlGRAS40* plants showed pleiotropic phenotypes, such as dwarfism, delayed flowering, reduced flower number, and decreased fruit set ratio [42, 43]. In addition, overexpression of *SlGRAS40* enhanced drought and salt tolerance in tomato [42]. By far, there are only seven GRAS proteins that have been functionally studied in tomato, including SlGRAS2, SlGRAS6, SlGRAS24, SlGRAS26, SlGRAS40, SlLs, and SlDELLA. It has been reported that GRAS proteins have multiple functions in many other plant species, so it is important to study the role of other GRAS proteins in tomato, which has not been functionally described yet. Here, we studied the functional description of *SlGRAS7* (accession number: Solyc07g065270.1.1), which belongs to a typical PAT1 subfamily gene. To further study the function of *SlGRAS7* in tomato, we constructed an overexpression vector to produce *SlGRAS7* upregulated transgenic lines. In this study, we found that overexpression of *SlGRAS7* resulted in pleiotropic phenotypes and enhanced drought and salt resistance. By evaluating gene expression and hormone responsiveness, we found that alterations in gibberellin and auxin signaling are likely to affect the substandard development of the overexpression of *SlGRAS7*.

## 2. Materials and Methods

### 2.1. Plant Growth Conditions

Tomato plants (*Solanum lycopersicum* cv. Micro-tom) were grown on soil (peat composite: vermiculite, 1 : 1) in 18 h light : 6 h dark cycles, 25°C day : 18°C night temperatures, and 60% relative humidity in controlled greenhouse conditions. The plants were treated with water-soluble fertilizers (Stanley Agriculture Group Co. Ltd) weekly. Different tissues from one-month-old WT plants, including leaves, roots, stems, flowers at the anthesis stage, fruits at the immature green, mature green, breaker, breaker plus one day, and orange and red stages, were collected for gene expression analyses. Samples were taken for each tissue from a minimum of seven plants. The different samples from plants were assorted and directly frozen in liquid nitrogen.

### 2.2. Vector Construction and Plant Transformation

The sequence of *SlGRAS7*, which does not have a stop codon, was amplified from the tomato cDNA and cloned into an expression vector. Using the standard method [44], *Agrobacterium tumefaciens* strain GV3101 was prepared to transfer the expression vector using the CaMV 35S promoter. In addition, *Agrobacterium tumefaciens* was used for transformation into WT tomato plants. Murashige and Skoog (MS) culture medium containing kanamycin was used to screen the positive transgenic lines. Eight *SlGRAS7* transgenic overexpression lines (OE) were produced. After qPCR analysis, three of eight homozygous transgenic lines (L1, L2, and L3) in T2 generation were selected for further experiments.

### 2.3. Gene Expression Analysis

Total RNA was isolated using an OMEGA BIO-TEK plant RNA kit. The RNA concentration and integrity were measured using a NanoDrop 1000 (Thermo, USA) and agar gel electrophoresis, respectively. First-strand cDNA synthesis was completed using a PrimeScript™ RT reagent kit with gDNA Eraser (TAKARA, Japan). A Bio-Rad CFX system (Bio-Rad, United States) was used for real time-qPCR with SYBR Green PCR Master Mix (CWBIO, China) in a 25 *μ*L total sample volume (1 *μ*L of primers, 1 *μ*L of cDNA, 10.5 *μ*L of distilled H_2_O, and 12.5 *μ*L of 2x SYBR Mix Taq). The RT-qPCR reactions were performed in a 96-well iCycler (Bio-Rad), with a temperature program starting with 3 min at 95°C, then 40 cycles of 5 sec at 95°C and 30 sec at 60°C. In the end, the melting temperature of the product was determined to verify the specificity of the amplified fragment. Three replicates were conducted for all samples. The *SlUBI* gene was used as an internal control. Relative expression levels were calculated based on the 2^-ΔΔCT^ method. All of the RT-PCR primers are shown in Supplementary Materials (Table S1).

### 2.4. Hormone Treatment for Plant Growth Analysis

GA_3_ (20 *μ*M) was sprayed on 10-day-old WT and *SlGRAS7*-OE L2 plants. Both genotypes were sprayed every 2 days for 4 weeks. Control WT and *SlGRAS7*-OE L2 plants were sprayed with water. The height of the plants and the flowering times for both WT and *SlGRAS7*-OE L2 were recorded.

Experiments for auxin dose-responses were conducted on one-week-old WT and *SlGRAS7*-OE L2 seedlings. The hypocotyl section (8 mm) under the cotyledon nodes was removed. The sections of the hypocotyl were placed in MES buffer/sucrose (5 mM MES/KOH, 1% (*w*/*v*) sucrose, and pH 6.0) and preincubated for 2 h. Hypocotyl sections were transferred into buffer solutions without or with NAA. After 23 h of incubation, the hypocotyl sections were measured at room temperature [45].

T2 transgenic lines of *SlGRAS7*-OE and WT seeds were sterilized, and the *SlGRAS7*-OE and WT seeds were embedded in the sterilized water for 3 days. The seeds were grown on an MS/2 medium with altered concentrations of GA_3_ (0, 0.5, 10, and 20 *μ*M). The seedlings were germinated in the light for 18 h and the dark for 6 h in a growth chamber. The day temperature was 25°C, and the night temperature was 20°C. The root and shoot lengths and the total plant height were measured after 15 days. Three replicates were performed on 25 plants for each experiment.

### 2.5. Hormone Treatment for Gene Expression Analysis

In the first experiment, fifteen-day-old WT seedlings were transferred into an MS/2 liquid medium for 0, 1, 3, 6, 12, or 24 h. In the second experiment, fifteen-day-old *SlGRAS7*-OE L2 and WT seedlings were transferred into an MS/2 liquid medium for 3 h. After treatment, the seedlings from both experiments were transferred into liquid nitrogen and stored at -80°C. The control samples were transferred into an MS/2 liquid medium without hormones. Each experiment was completed with three replicates.

### 2.6. Abiotic Stress Treatments

Plant leaves of one-month-old WT were sprayed with 100 mM D-mannitol and 200 mM NaCl to serve as the osmotic and salt stress treatments, respectively. The control WT plants were treated with water. The leaves from the control-, D-mannitol-, and NaCl-treated WT plants were collected after 1 h, 3 h, 6 h, 12 h, and 24 h. Leaves from six plants were collected for each sample and mixed well. Each sample was transferred into liquid nitrogen and stored at -80°C until RNA extraction.

To analyze the salt and drought tolerance, 15 WT plants and 15 plants from each line of *SlGRAS7*-OE (L1, L2, and L3) were grown in a large pot, and the pots were watered three times a week. The water was constant in all pots. All WT and *SlGRAS7*-OE plants (L1, L2, and L3) were grown at the same temperature and light conditions. After 2 weeks, the WT and *SlGRAS7*-OE plants (L1, L2, and L3) were treated as the control, salt, and drought treatments. For the salt treatment, the WT and *SlGRAS7*-OE (L1, L2, and L3) plants were watered with 200 mM NaCl at 2-day intervals for one month. The plants were treated without watering to analyze drought. The control plants were treated with water. Light and temperature conditions were the same for all plants treated. The relative water content and total chlorophyll [46] were tested after each treatment. Leaf samples were collected at the same developmental phase after salt and drought treatment, immediately frozen in liquid nitrogen and stored at -80°C until RNA extraction.

For salt and osmotic tolerance analysis, WT and *SlGRAS7*-OE (L1, L2, and L3) seeds were sterilized and sown on MS/2 alone or MS/2 containing 75 mM NaCl and 150 mM D-mannitol, respectively [47]. Control WT and *SlGRAS7*-OE seeds (L1, L2, and L3) were germinated on MS/2 without NaCl and D-mannitol. Seeds were germinated in a growth chamber with 18 h light (25°C) and 6 h darkness (18°C) cycles. The primary roots and hypocotyl lengths were measured after 15 days, and the rate of the seed germination was calculated after one week.

### 2.7. Statistical Analysis

Each experiment was conducted with three independent biological replicates. Student's *t*-test was used to compare group differences. *P* values less than 0.05 were considered to be significant.

## 3. Results

### 3.1. Phenotypic Characterization of the SlGRAS7-OE Transgenic Plants

**Figure 1:**
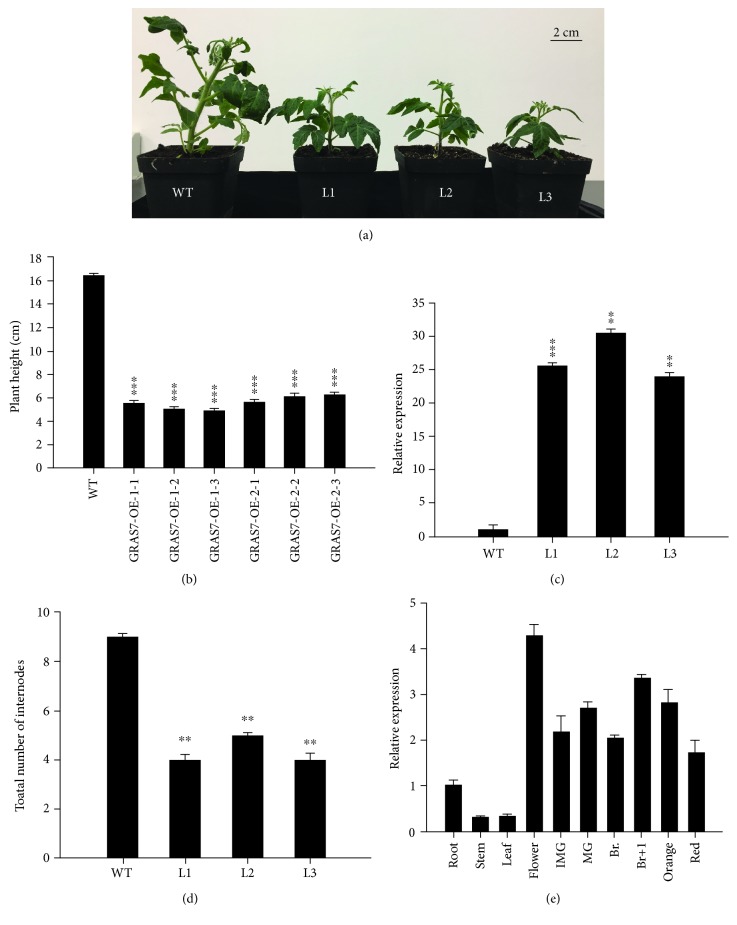
Phenotypic characterization of WT and *SlGRAS7-*OE. (a) One-month-old plants of WT and transgenic plants of *SlGRAS7*-OE lines L1, L2, and L3. (b) The height of plants WT and both generations (T1, T2) of *SlGRAS7*-OE shown in (a). Error bars show the standard error between three biological replicates (*n* = 3) with more than 20 plants for each replicate performed. (c) The expression level of *SlGRAS7* of one-month-old plants of WT and *SlGRAS7*-OE lines. Expression data were normalized with the *SlGRAS7* expression in WT as 1. Error bars show the standard error between three biological replicates (*n* = 3). (d) A total number of internodes of one-month-old plants of WT and *SlGRAS7*-OE (L1, L2, and L3). (e) Tissue profiling analysis of *SlGRAS7* in different organs of one-month-old wild-type plants. Expression data were normalized with the *SlGRAS7* expression in the root set as 1.

To evaluate the physiological significance of *SlGRAS7*, transgenic tomato plants expressing the *SlGRAS7* cDNA were produced using a CaMV 35S promoter by transformation with *Agrobacterium tumefaciens*. WT and overexpressing *SlGRAS7* plants are shown in Figure 1(a). Leaves from one-month-old plants of three independent lines, L1, L2, and L3, were found to overexpress the gene by 25.55-fold, 30.43-fold, and 23.95-fold, respectively (Figure 1(c)). Overexpression of the *SlGRAS7* plants resulted in pleiotropic phenotypes with dwarfism, delayed flowering time, and fewer fruits and seeds. Supplementary Materials (Table S2) show the additional details of the *SlGRAS7* phenotypes. *SlGRAS7* was expressed in all tissues of the WT examined. *SlGRAS7* was expressed at higher levels in the flowers and breaker+1 in the WT (Figure 1(e)).

### 3.2. SlGRAS7-OE Displays Altered Responsiveness to GA_3_ and IAA

**Figure 2:**
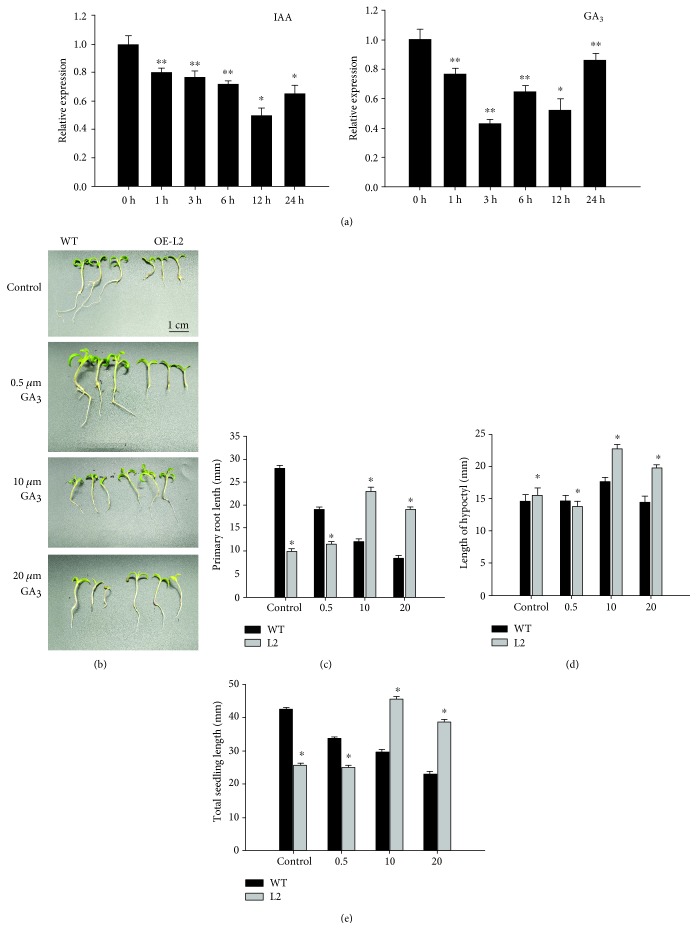
Overexpression of *SlGRAS7* alters responsiveness to GA_3_ and IAA. (a) Quantitative RT-PCR analysis of *SlGRAS7* from 15 days of WT seedlings treated with 20 *μ*M GA_3_ and 20 *μ*M IAA. (b) Phenotypes of 15 days of WT and *SlGRAS7*-OE L2 seedlings grown on an MS/2 medium containing (0 *μ*M, 0.5 *μ*M, 10 *μ*M, and 20 *μ*M GA_3_). (c) Primary root length of WT and *SlGRAS7*-OE seedlings shown in (b). (d) Hypocotyl length of WT and *SlGRAS7*-OE seedlings shown in (b). (e) Plant height of WT and *SlGRAS7*-OE seedlings shown in (b). Asterisks show the significant differences using Student's *t*-test (^∗^ *P* < 0.05, ^∗∗^ *P* < 0.01).

**Figure 3:**
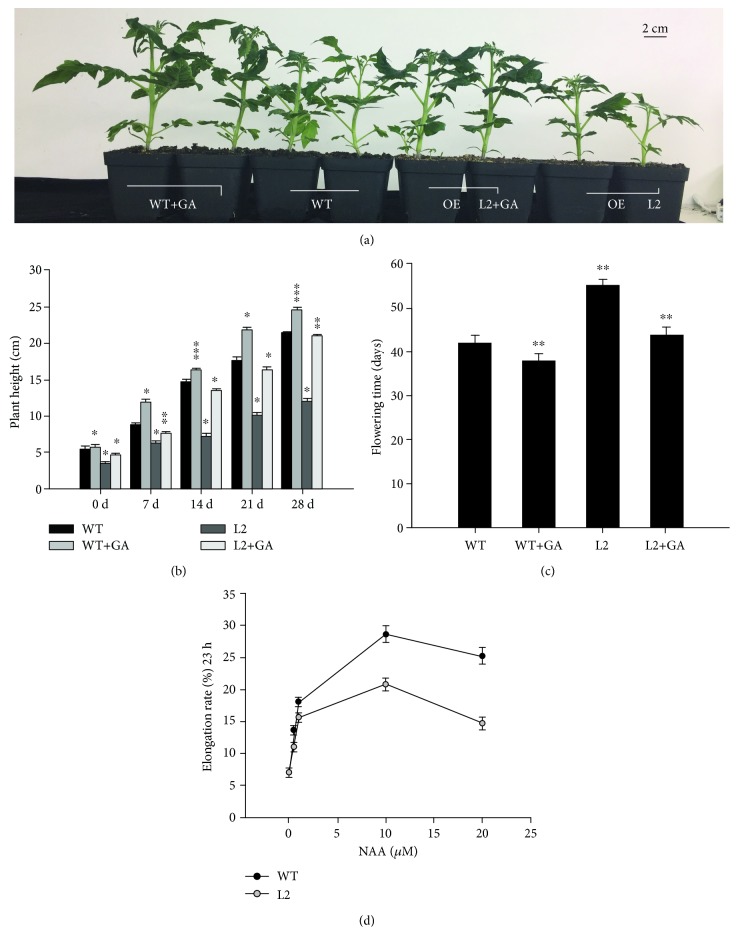
(a) Rescue of *SlGRAS7*-OE L2 dwarfism by the exogenous GA_3_ application. (b) Plant height and (c) flowering time of GA_3_-treated plants shown in (a). (d) Hypocotyl elongation of WT and SlGRAS7-OE L2 after NAA treatment. Asterisks show the significant differences using Student's *t*-test (^∗^ *P* < 0.05, ^∗∗^ *P* < 0.01).

The level of expression of *SlGRAS7* decreases after treatment with GA_3_ and IAA in WT (Figure 2(a)), which indicates that *SlGRAS7* responds to gibberellin and auxin. To study the functions of *SlGRAS7* in response to the phytohormone GA_3_, WT and *SlGRAS7*-OE L2 seedlings were germinated with altered concentrations of GA_3_ (0 *μ*M, 0.5 *μ*M, 10 *μ*M, and 20 *μ*M). The seedlings that overexpressed *SlGRAS7* had no lateral roots and longer primary roots as well as longer hypocotyls than the WT after GA_3_ treatment (data of L2 in Figures 2(c)–2(e), data of L1 in Figure S1), indicating that overexpression of *SlGRAS7* changes the responsiveness to GA_3_. These data showed that *SlGRAS7*-OE reduced the responsiveness of the hypocotyl to GA_3_. The lengths of the roots and hypocotyls of *SlGRAS7*-OE L2 seedlings were more elongated than those of the WT and *SlGRAS7*-OE L2 not treated with GA_3_ in response to 10 *μ*M and 20 *μ*M GA_3_. The primary root lengths of *SlGRAS7*-OE L2 seedlings were smaller than WT seedlings under the control and 0.5 *μ*M (Figure 2(b)). The phenotype of the small height and delayed flowering time of *SlGRAS7*-OE plants could be controlled by exogenous application of 20 *μ*M GA_3_, and their levels became parallel with those of the WT (Figure 3). Thus, the results indicated that *SlGRAS7* is involved in GA signaling or biosynthesis. The overexpression of *SlGRAS7* showed auxin sensitivity, which was determined by the enlargement of the hypocotyl sections under the auxin dose assay. At all concentrations of auxin, the hypocotyl of *SlGRAS7*-OE L2 was shorter than that of the WT, but the greatest amount of enlargement of the hypocotyl was observed at 10^−5^ M NAA concentrations in the WT, as well as in *SlGRAS7*-OE L2 (Figure 3(d)). These results showed that overexpression of *SlGRAS7* is involved in the reduction of hypocotyl auxin sensitivity.

### 3.3. SlGRAS7-OE Enhances Tolerance under Salt and Drought Stress

**Figure 4:**
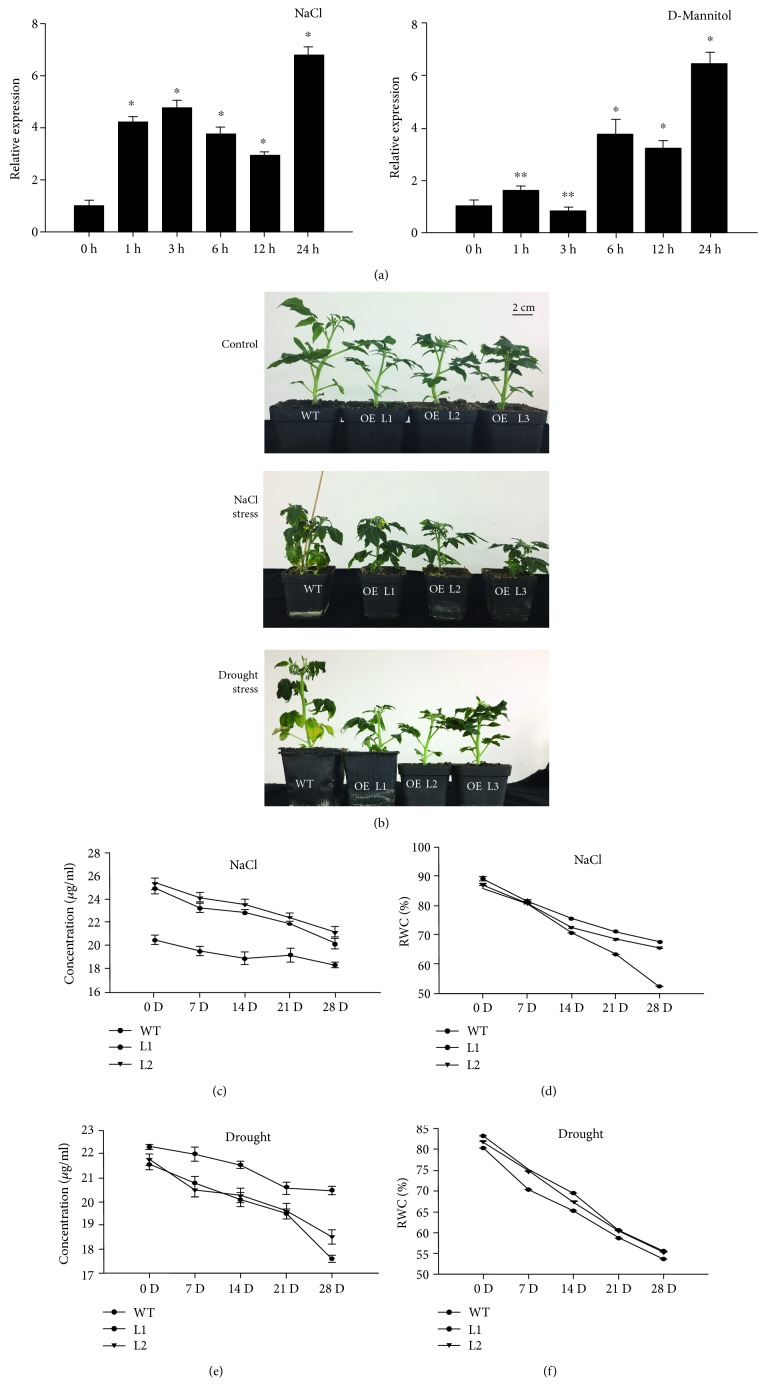
Overexpression of *SlGRAS7* enhances tolerance to salt and drought stress treatment. (a) Quantitative RT-PCR analysis of *SlGRAS7* from one-month-old plants sprayed with 200 mM NaCl and 100 mM D-mannitol. Expression data were normalized with an expression of *SlGRAS7* in treated plants as 0 h set as 1. Asterisks show significant differences using Student's *t*-test (^∗^ *P* < 0.05, ^∗∗^ *P* < 0.01). (b) Photographs of representative plants after one month of NaCl salt treatment and of drought stress compared to control plants. (c, d) Total chlorophyll concentration and relative water content (RWC) of plants shown in (b) under salt stress. (e, f) Total chlorophyll concentration and relative water content (RWC) of plants shown in (b) under drought stress.

WT plants were treated with NaCl and D-mannitol to show the saline and osmotic effects, respectively. *SlGRAS7* was highly upregulated in response to both 200 mM NaCl and 100 mM D-mannitol stress (Figure 4(a)). Therefore, *SlGRAS7* may be involved in the abiotic and osmotic stress response in tomato.

To explore the role of *SlGRAS7* under salt and drought stress, WT plants and *SlGRAS7*-OE L1, L2, and L3 were treated with a solution of 200 mM NaCl every 48 h for up to one month to examine salt stress tolerance. WT and *SlGRAS7*-OE L1, L2, and L3 plants were deprived of water for up to one month to examine drought stress. Under NaCl salt stress and drought stress treatment, all *SlGRAS7*-OE plants were healthier than the WT (Figure 4(b)). After one month, WT plants generally displayed more necrosis and chlorosis under salt stress treatment, while there was no apparent damage to *SlGRAS7*-OE plants (Figure 4(b)). Under drought stress treatment, the lower leaves wilted more in WT plants, but the only insignificant damage was noted in *SlGRAS7*-OE plants (Figure 4(b)).

During salt and drought stress treatments, both the relative water content (RWC) and the total chlorophyll content reduced in WT and *SlGRAS7*-OE plants, while the levels of both were much greater in *SlGRAS7*-OE plants than in WT (Figures 4(c)–4(f)).

### 3.4. SlGRAS7-OE Enhances the Seed Germination Rate under Salt and Osmotic Stress

**Figure 5:**
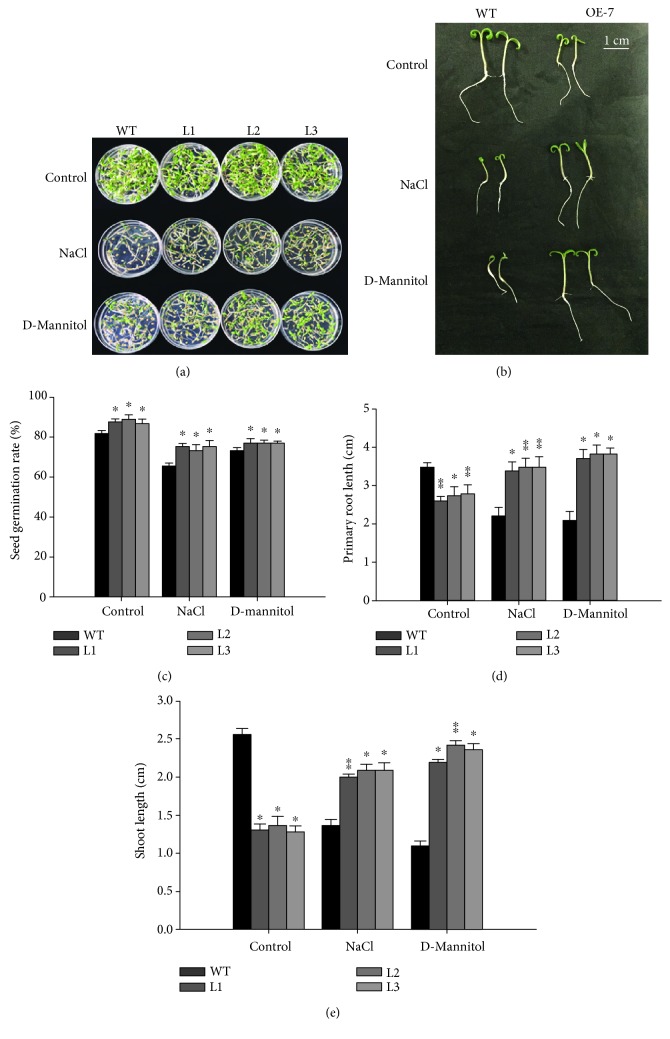
Comparative analysis of *SlGRAS7*-OE under salt and osmotic stress. (a, b) Seed germination of WT and *SlGRAS7*-OE under salt and D-mannitol stress treatments after 2 weeks. (c) The germination rate of WT and *SlGRAS7*-OE lines under control, salt, and osmotic stress. (d) Primary root length of WT and *SlGRAS7*-OE under control, NaCl, and D-mannitol treatments. (e) Shoot lengths of WT and *SlGRAS7*-OE under control, NaCl, and D-mannitol treatments. Error bars indicate the standard errors between three replicates. Asterisks show significant differences using Student's *t*-test (^∗^ *P* < 0.05, ^∗∗^ *P* < 0.01).

WT and *SlGRAS7*-OE L1, L2, and L3 plants were examined to determine the salt and osmotic tolerance of seed germination (Figure 5(a)). The rate of seed germination of both WT and *SlGRAS7*-OE decreased in response to 75 mM NaCl and 150 mM D-mannitol, respectively (Figure 5(a)), but the rate of seed germination of *SlGRAS7*-OE was much greater than that of WT seeds in response to both stress treatments. The average rate for the salt treatment was 65%/73.8% and 76.7%/72.4% for the osmotic treatment. Root elongation was affected in response to salt and osmotic stress treatments, and the root length of *SlGRAS7*-OE was longer than that of the WT under the salt and osmotic treatments (Figure 5(c)). The shoot lengths of *SlGRAS7*-OE were larger than those of the WT under both stress treatments (Figure 5(d)). The root and shoot lengths of WT were significantly reduced under both stress treatments. These results indicate that the seeds and seedlings of *SlGRAS7*-OE tolerate salt and osmotic stress.

### 3.5. Expression Analysis of Auxin- and GA-Related Genes in SlGRAS7-OE Plants

**Figure 6:**
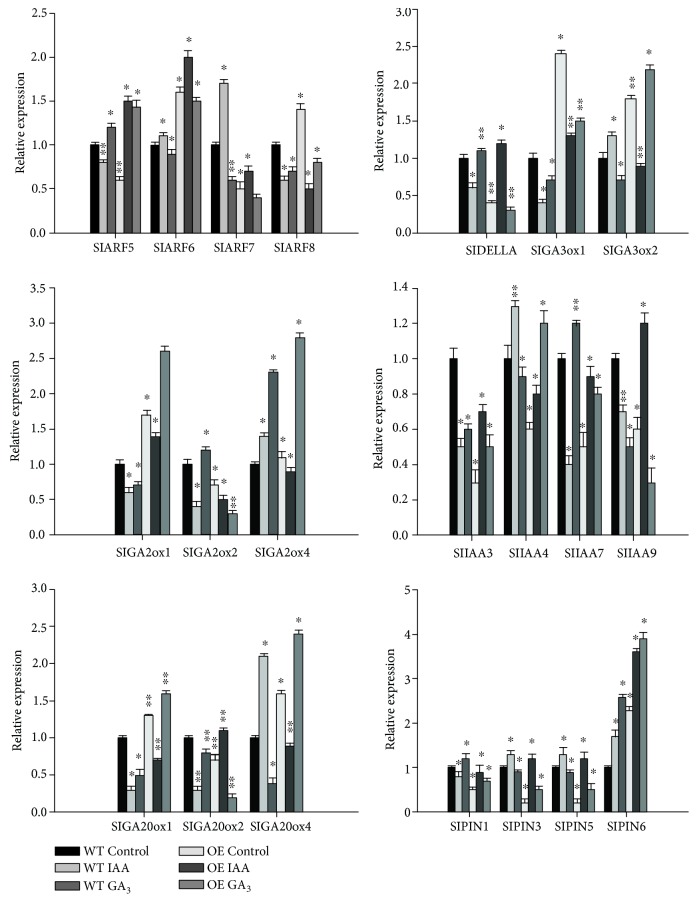
Expression analysis of auxin/GA-related genes. qRT-PCR analysis of auxin and GA-related genes in 15 days old WT and *SlGRAS7*-OE seedlings as well as in response to IAA and GA_3_ treatment (20 *μ*M for 3 h). Error bars represent the standard error between three biological replicates performed. Asterisks show significant differences using Student's *t*-test (^∗^ *P* < 0.05, ^∗∗^ *P* < 0.01).

To examine the role of *SlGRAS7* in the auxin and GA pathways, the levels of expression of 21 tomato genes were tested in WT seedlings and those of *SlGRAS7*-OE L2 under auxin and GA_3_ treatments (Figure 6). Four PIN-FORMED (PIN) auxin efflux transport proteins (*SlPIN1*, *SlPIN3*, *SlPIN5*, and *SlPIN6*), four auxin response gene (ARF) transcription factors (*SlARF5*, *SlARF6*, *SlARF7*, and *SlARF8*), three GA deactivating enzymes (*SlGA2ox1*, *SlGA2ox2*, and *SlGAox4*), four indole-3-acetic acid/auxin (IAA/Aux) transcription factors (*SlIAA3*, *SlIAA4*, *SlIAA7*, and *SlIAA9*), a key regulator of the GA signaling pathway (*SlDELLA*), and five GA biosynthetic enzymes (*SlGA20ox1*, *SlGA20ox2*, *SlGA20ox4*, *SlGA3ox1*, and *SlGAox2*) were examined to determine their expression levels in the WT and *SlGRAS7*-OE. In the control, nine genes showed higher expression and 12 showed lower expression in the *SlGRAS7*-OE, which indicated that overexpression of *SlGRAS7* altered auxin and GA homeostasis in overexpressing plants. In addition, when these results were compared to WT, some genes showed different responses to IAA and GA_3_ in *SlGRAS7*-OE seedlings. For example, the expression of *SlAFR5* is downregulated in the WT but upregulated in the *SlGRAS7*-OE seedlings in response to IAA treatment. *SlPIN6* was upregulated by IAA and GA_3_ in the WT and *SlGRAS7*-OE. *SlDELLA* was downregulated by IAA but upregulated by GA_3_ in the WT, but it was upregulated under the IAA treatment and downregulated under the GA_3_ treatment in *SlGRAS7*-OE. GA_3_ induces the upregulation of *SlGA2ox4* in both WT and *SlGRAS7*-OE. Without hormone treatment, the expression of *SlGA20ox1* was upregulated in *SlGRAS7*-OE seedlings, but IAA treatment caused the downregulation of expression. WT and *SlGRAS7*-OE seedlings displayed a dramatic response to the IAA-related genes during GA_3_ treatment. In contrast, the GA-related genes during IAA treatment could indicate that *SlGRAS7* acts as an integrator between the auxin and GA pathways. However, it is likely that *SlGRAS7* has a role in the regulation of hormone-related gene expression in tomato, primarily in the genes related to auxin and GA transport, biosynthesis, and signal transduction.

### 3.6. Expression Analysis of Stress-Related Genes in WT and SlGRAS7-OE Plants under Salt and Drought Stress

**Figure 7:**
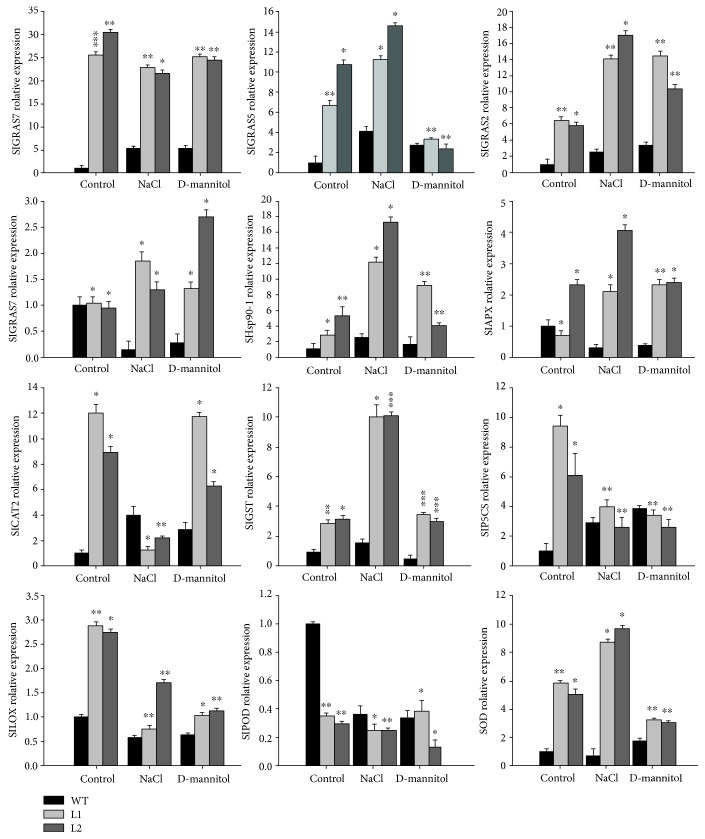
Expression levels of stress-related genes (*ERF1*, *ERF5*, *GME2*, *Hsp90-1*, *P5CS APX*, *CAT2*, *GST*, *LOX*, *POD*, and *SOD*) in WT and *SlGRAS7*-OE (L1, L2) under normal, NaCl, and D-mannitol. Error bars show the standard error of data. Asterisks show the significant differences using Student's *t*-test (^∗^ *P* < 0.05, ^∗∗^ *P* < 0.01).

Quantitative reverse transcription- (qRT-) PCR was used to examine the expression of plant stress response biomarkers to determine the molecular mechanisms involved in the enhanced resistance of *SlGRAS7*-OE in response to salt and drought stress (Figure 7). Scavenging and ROS generation alter the transcript levels of many genes involved, such as *CAT*, *POD*, *SOD*, ascorbate peroxidase (*APX*), glutathione S-transferase (*GST*), and lipoxygenase (*LOX*). These were measured under normal and stress conditions in both WT and *SlGRAS7*-OE. In *SlGRAS7*-OE plants, the expression levels became higher than those in WT under stress conditions (Figure 7). An ascorbic acid synthetase gene (*SlGME2*) showed upregulation in response to the control and stress conditions in *SlGRAS7*-OE plants compared to WT, and the level of *SlGEM2* also increased under stress conditions in WT plants (Figure 7). A heat shock protein (*SlHsp90-1*) had higher levels of *SlGRAS7*-OE plants than in WT after salt and drought treatment (Figure 7). An ethylene-responsive factor (*SlERF1*), an ethylene-responsive LEA protein (*SlERF5*), and an ascorbate peroxidase gene (*SlAPX*) all had higher levels in the *SlGRAS7*-OE plants under control and salt conditions compared to that in the WT (Figure 7). *SlCAT2* was upregulated after control and drought conditions in both *SlGRAS7*-OE plants and WT plants, but after salt stress, the transcript level decreased more in *SlGRAS7*-OE plants than in WT plants (Figure 7). These results showed that *SlGRAS7* could play an important role in stress signaling pathways by modifying these genes in tomato.

## 4. Discussion

Mounting evidence shows that GRAS transcription factors play dynamic roles in plant development and signal transduction pathways. A comprehensive studied miR171-GRAS control network takes part in complex physiological developments, such as shoot branching, shoot meristem maintenance, trichome distribution, chlorophyll biosynthesis, and flowering time [26, 27, 43, 48]. Recently, its similar regulatory module has been studied in tomato [42, 43]. Overexpression of *SlGRAS40* enhances tolerance to abiotic stresses and influences gibberellin and auxin pathway during reproductive and vegetative growth in tomato [42], and overproduction of a tomato miR171 target gene *SlGRAS24* impacts several agronomical behaviors through regulating auxin and gibberellin homeostasis [43]. Downregulation of *SlGRAS26* altered plant phase transition and morphological traits in tomato. *SlGRAS26* showed a response to ABA, GA, IAA, dehydration, and abiotic stresses [49]. There are eleven GRAS proteins that belong to the PAT1 subfamily. However, the function of PAT1 branch has never been described so far in tomato. Here, one gene from the PAT1 subfamily, *SlGRAS7*, was functionally recognized. *SlGRAS7* showed enhance resistance to abiotic stresses and hormone treatments. These results indicated that *SlGRAS7* may be involved in the abiotic stress responsive and mediating hormone signaling.

Salt and drought stress can lower metabolic reactions, reduce photosynthetic capacity, and enhance the oxidative loss of cells [50]. Due to salt and drought stress, indications of damage to the plants, such as necrosis, chlorosis, and wilting, were all delayed in overexpressing *SlGRAS7* tomato plants compared to WT tomato plants (Figure 4(b)). The concentration of total chlorophyll and the relative water content were higher in *SlGRAS7*-OE plants than in WT (Figures 4(c)–4(f)). Under NaCl and D-mannitol stress treatments, the germination rates of seeds and seedlings were less affected in *SlGRAS7*-OE than in WT (Figure 5(b)). The results showed that *SlGRAS7*-OE increased the ability to resist salt and drought stress during vegetative growth. Several genes have been reported to be upregulated in the vegetative tissues in response to stress treatments [51, 52]. In this study, the transcription levels of numerous genes have been confirmed to affect ROS scavenging (Figure 7). Under control and stress treatments, the expression of *SlSOD*, *SlLOX*, *SlGST*, *SlCAT2*, and *SlAPX* increased during the overexpression of *SlGRAS7* compared to the WT (Figure 7). *SlERF1*, a key factor of biotic/abiotic stress responses [53], and *SlERF5* showed higher levels of expression in *SlGRAS7*-OE under salt and drought stress conditions compared to WT plants (Figure 7). SlGME2, an important catalytic enzyme in the biosynthesis of ascorbic acid [54], and heat shock protein (SlHsp90-1) both showed higher expression under salt and drought stress. These results indicated that *SlGRAS7*-OE modifies gene expression involved in stress signaling pathways, which could be a mechanism to increase salt and drought stress tolerance. In contrast, *SlGRAS7*-OE resulted in the adaptation of various significant agronomical behaviors, including plant height, stem length, stem diameter, leaf length, leaf diameter, and flowering time (Supplementary Table S2), which makes it a good target gene to produce varieties with differing plant architectures and flowering times that result in altered yields. Thus, this could result in varieties that help achieve the maximum demand for nutrition, feed, and biofuel production.

Gibberellin and auxin participate in abiotic stress responses in plants. For instance, ABA signaling and salt-activated ethylene pathways integrate at the level of DELLAs to enhance salt resistance [55]. Under abiotic stress, DELLA proteins are also involved in the regulation of growth and ROS reactions [36]. Mannitol and salt stress treatments can boost the accretion of DELLAs with upregulation of the genes encoding antioxidant mechanisms, supersede with the cutback in ROS abundance [55, 56]. An association has been found between abiotic stress and endogenous auxin levels in rice in which overexpression of *OsGH3.13* or *OsPIN3t* enhanced resistance under drought [57, 58]. Various studies also showed that there is a correlation between ROS and auxin pathways. The exogenous application of auxin reduced the H_2_O_2_ content in the roots of the tomato by enhancing the activity and expression of H_2_O_2_ scavenging enzymes [59].

Several studies showed that GAs control various developmental and growth processes, for instance, stem elongation [60]. An *ERF/AP2* transcription factor, overexpression of *SlDREB*, shows small heightened tomato plants by inhibiting gibberellin biosynthesis to decrease endogenous gibberellin level [61]. Overexpression of *AtGA20ox1*, *AtGA20ox2*, and *AtGA20ox3* augmented shoot growth and exhibited elongated hypocotyls by producing more dynamic GAs in *Arabidopsis* [34, 62]. Overexpression of the *CcGA20ox1* gene in tomato also has similar phenotypes [63]. Moreover, GRAS proteins have been reported in association with GA regulation. For instance, GRAS protein SCL3 and DELLA antagonize each other in controlling both downstream GA responses and upstream GA biosynthetic genes [33]. The association of GRAS proteins and GA has been widely known [19]. However, some proteins from the GRAS family have also been known to be involved in auxin signaling. For example, AtSCL15 is an auxin-induced GRAS protein involved in seed maturation [64]. By modulating both auxin and GA signaling, the SHR/SCR complex has been shown to participate in root growth [32, 33, 37]. The relation between auxin and GA has been clearly shown by the identification of crosstalk and self-regulatory pathways, including genes related to GA metabolism and auxin transport. However, numerous significant characteristics of this relationship are still unknown. Plant hormones function in the development and growth of root apical meristems (RAMs) and shoot apical meristems (SAMs) [65–67]. Auxin/GA signaling roles have been recognized in RAMs and SAMs [68]. It has been shown that *HAM* genes are necessary to sustain both RAMs and SAMs [69], indicating that these genes could play roles in RAMs and SAMs by controlling auxin and GA signaling. While *Atham1*, *2*, *3* is known to produce root apex auxin maxima that are related to the WT in intensity and spatial expression in Arabidopsis [69], it did not directly correlate with the action of the *AtHAM* gene to auxin signaling. In this study, we show that SlGRAS7 is an important transcription factor that may be involved in auxin and GA signaling pathways. It is also known that a GRAS-like gene of sunflower altered the gibberellin content and axillary meristem outgrowth of transgenic *Arabidopsis* plants [70]. The overexpression of *SlGRAS7* in tomato plants resulted in a dwarf phenotype that has small primary roots, short stem lengths, and later flowering time (Supplementary Table S2). Some GRAS has been shown to function as regulators of gibberellin and auxin in the development and growth of the plant. It has been described how *SlGRAS24* influenced a number of agronomical behaviors in tomato by regulating gibberellin and auxin homeostasis [43]. In our study, overexpression of *SlGRAS7* altered the responsiveness to GA_3_ and IAA (Figure 2), which leads to a shortage of GA, auxin insensitivity, and an altered abundance of transcripts linked to gibberellin and auxin signaling and biosynthesis (Figure 6). Some of the GA-related genes were downregulated, indicating that the GA content was disrupted in *SlGRAS7*-OE (Figure 6). In addition, the application of GA_3_ partially inhibited the dwarf phenotype and the growth rate to the WT level in *SlGRAS7*-OE plants (Figures 3(a)–3(d)). These results suggested that *SlGRAS7* may be involved in GA and auxin signaling. Our results also indicated that *SlGRAS7* may enhance the abiotic stress response via GA/auxin signaling in tomato plants. *SlGRAS7* disturbs auxin signaling and represses gibberellin biosynthesis by reducing gene expression encoding auxin transporters and receptors, and GA biosynthesis stimulating enzymes, respectively, then affects auxin and gibberellin homeostasis. Consequently, we conclude that overexpressing *SlGRAS7* plants may enhance the abiotic tolerance and ROS scavenging ability. Also, the crosstalk between gibberellin and auxin may stimulate DELLA accumulation under abiotic stresses in *SlGRAS7*-OE plants. Thus, our studies on *SlGRAS7* shows that GRAS may play an integrative function in tomato and may enhance tolerance to abiotic stresses, gibberellin, and auxin signaling during reproductive and vegetative growth.

## Data Availability

The data used to support the findings of this study are included within the article.

## Abbreviations

GRAS: Gibberellic acid insensitive, repressor of GA1, and scarecrow
WT: Wild-type
SlGRAS7-OE: Overexpression of SlGRAS7
GRNs: Gene regulatory networks
TF: Transcription factors
LHR: Leucine heptad repeat
GA: Gibberellin
IAA: Indole acetic acid
cDNA: DNA complementary to RNA
RNA: Ribonucleic acid
RT-qPCR: Real-time quantitative PCR
FAA: Formalin-acetic-alcohol
bZIP: Basic-domain leucine-zipper
MYC/MYB: Myelocytomatosis/myeloblastosis
NAC: N-Acetylcysteine
Dof: DNA-binding with one finger
LOM: Lost meristem
SPL: Squamosa-promoter binding protein-like
SCL: Scarecrow-like
MES: 2-(N-morpholino) ethanesulfonic acid
KOH: Potassium hydroxide
MS: Murashige and Skoog
NaCl: Sodium chloride
RWC: Relative water content
ROS: Reactive oxygen species
ABA: Abscisic acid
ARF: Auxin response factor
IAA/Aux: Indole-3-acetic acid/auxin
CAT: Catalase
POD: Peroxidase
SOD: Superoxide dismutase
APX: Ascorbate peroxidase
GS: Glutathione S-transferase
LOX: Lipoxygenase
GME: GDP-mannose 3 ′,5 ′-epimerase
Hsp: Heat shock protein
ERF: Ethylene-responsive factor
LEA: Late embryogenesis abundant
APX: Ascorbate peroxidase
H_2_O_2_: Hydrogen peroxide
SHR/SCR: Short root/scarecrow
RAM: Root apical meristem
SAM: Shoot apical meristem
HAM: Hairy meristem.

## Supplementary Materials

Supplementary Materials Supplementary Table S1: primers used for qRT-PCR. Supplementary Table S2: phenotypes of WT and *SlGRAS7*-OE plants. Supplementary Figure S1: 15 days of WT and *SlGRAS7*-OE L2 seedlings grown on an MS/2 medium containing (0 *μ*M, 0.5 *μ*M, 10 *μ*M, and 20 *μ*M GA_3_). (a) Primary root length of WT and *SlGRAS7*-OE (L1) seedlings. (b) Hypocotyl length of WT and *SlGRAS7*-OE (L1) seedlings. (c) Plant height of WT and *SlGRAS7*-OE seedlings (L1). Asterisks show the significant differences using Student's *t*-test (^∗^ *P* < 0.05, ^∗∗^ *P* < 0.01).
