# Supplementary material for: Overexpression of SlGRAS7 Affects Multiple Behaviors Leading to Confer Abiotic Stresses Tolerance and Impacts Gibberellin and Auxin Signaling in Tomato
Source: Int J Genomics. 2019 Jul 1;2019:4051981. doi: 10.1155/2019/4051981 (PMC6636567; doi:10.1155/2019/4051981)
Supplement: Supplementary Materials — Supplementary Table S1: primers used for qRT-PCR. Supplementary Table S2: phenotypes of WT and SlGRAS7-OE plants. Supplementary Figure S1: 15 days of WT and SlGRAS7-OE L2 seedlings grown on an MS/2 medium containing (0 μM, 0.5 μM, 10 μM, and 20 μM GA3). (a) Primary root length of WT and SlGRAS7-OE (L1) seedlings. (b) Hypocotyl length of WT and SlGRAS7-OE (L1) seedlings. (c) Plant height of WT and SlGRAS7-OE seedlings (L1). Asterisks show the significant differences using Student's t-test (∗ P < 0.05, ∗∗ P < 0.01). [file 4051981.f1.pdf]

International Journal of Genomics

Overexpression of *SlGRAS7* affects multiple behaviors leading to confer abiotic stresses tolerance and impacts gibberellin and auxin signaling in Tomato

Sidra Habib[1], Muhammad Waseem[1], Ning Li[1], Lu Yang[1], and Zhengguo Li[1,*]

[1] School of Life Science, Chongqing University, Chongqing, 400044, P.R. China.

Correspondence should be addressed to Zhengguo Li; L1400065@cqu.edu.cn

Table S1. Primers used for qRT-PCR

	Primers for qRT-PCR	
Gene Name	Forward (5'-3')	Reverse (5'-3')
SlGRAS7 (Solyc07g065270.1.1)	ATGAGGCCTTCTCAGAACCCTGAGG	TTTAGTTGCTTCTTGTGCATGGAAA
SlIAA3 (Solyc09g065850)	CTCAGGAATGTATTTAAAAGTTAG	TCCTTCTCTTTCTGAATACACT
SlIAA4 (Solyc06g053840)	AACAAGAGGGCTTTGCCTGAG	GTGTCTTGGCAACAGGTGGA
SlIAA7 (Solyc06g053830)	ACTCAACCTCCATCATAATGATAATATTCC	ACCCCACCACTTGAGCCTTA
SlIAA9 (Solyc04g076850)	CCCCTTGCACCCTTCCA	AGCGTCTGAAAATCCTCGTTTG
SlARF5 (Solyc04g081240)	ATTAGTTCTGAGTTGTGGC	GGTATCTGTGAAGTTGCTG
SlARF6 (Solyc07g043460)	GGTTCAACGGGTTCTCAAC	TTCAGGGAAGTGGATGCTC
SlARF7 (Solyc07g042260)	CCAAGTTATCCTAATCTTCCTTCC	GTAAAGCCTCCTGGTCATATTTG
SlARF8 (Solyc02g037530)	CTGCTCAAACCCAAATGCTGTC	GGTAAGTGTGTTGGTGAGCCTG
SlPIN1 (Solyc03g118740)	GCTGCAGGCTGGTCTAGATT	AACAATGGCAACAAAGCACA
SlPIN3 (Solyc04g007690)	TTCAAAATCAATTTAGCGTGTCA	CTCAAAATCCCTCTTGTTTCG
SlPIN5 (Solyc01g068410)	ACATTGAGCTGGCATTTTGG	TCCACTACCAGCCTTTGACA
SlPIN6 (Solyc06g059730)	AGATGGCAGCAATAGGGATG	GCGAAGACAAATGGAACGAT
SlGA20ox1 （Solyc03g006880）	CTCATTTCTAATGCTCATCGT	TGCAGATGATTCTTTCTTAGCG
SlGA20ox2 （Solyc06g035530）	TTTCCATATTCTACCCTACAAG	TCATCGCATTACAATACTCTT
SlGA20ox4 （Solyc01g093980）	GATGATAAATGGCACTCTATTC	TGACTTCCTTGTTCTTCTACAG
SlGA3ox1	GGCATTAGTAGTTAATATAGGTGA	AAATAAGCTACAGAAAGTCGATA

（Solyc06g066820）		
SlGA3ox2 （Solyc03g119910）	GATCATAAATTTGTCATGGATAC	TGTTTCCATATGGTTAAGTAATC
SlGA2ox1 （Solyc05g053340）	GGCATGTAAGATATTAGAATTGA	TTAATCCGTAGTAGAGAATCAGA
SlGA2ox2 （Solyc07g056670）	ATTAAGATCCAATAACACTTCG	TCTTGATTTCACACTATTTGC
SlGA2ox4 （Solyc07g061720）	ATGGAAGGAAAAGACAGTTTA	CTTTTCTCAAATAGGACCAAC
SlDELLA （Solyc11g011260）	TGATGCGACTATACTTGATATAAG	GGGTTAATCTGTTTAATAGAGTTC
SlUBI (Solyc07g064130)	GCCGACTACAACATCCAGAAGG	TGCAACACAGCGAGCTTAACC
SlCAT (Solyc12g094620)	AAGTCCTGTGGTCAGAAGGTCG	GAAGTACAGTTTATAGCACAACGCG
SlSOD (Solyc01g067740)	TGAATTGGGGTTGAACCATT	GCAGGCACTGTAATCTGCAA
SlPOD （Solyc11g018800）	CTTGCCCTAATGCTCTCACC	GCATCACAACCCTGAACAAA
SlGST (Solyc09g011590)	GCAAGCCCATTTGTGAGTCT	TGCTGACCCCTTATCATCG
SlLOX (Solyc01g006560)	TCATTTTCCCCTGGCAAGTA	TGGTGCATTTGGATCTTCCT
SlAPX (Solyc06g005160)	ACGATGATATTGTGACACTCTTCCA	AAGCGATGAAACCACAAAAACA
SlP5CS (Solyc08g043170)	TGCTGTAGGTGTTGGTCGTCA	TGCCATCAAGCTCAGTTTGTG
SlERF1 (Solyc03g093610)	TTTTAGTATCGGATGGACG	GGCGGAGAAACAGAAGTA
SlHsp90-1 (Solyc12g015880)	GCACTTCTCTGTTGAAGGTCAG	ATGAACACACGGCGAACATA
SlGEM2 (Solyc09g082990)	CCATCACATTCCAGGACCAGA	CGTAATCCTCAACCCATCCTTC
SlER5 (Solyc01g095140)	TATTGGTAAAGATTGGGACATTGA	TGTCTTCTTGTTTGTCACCGTTC

Table S2. Phenotypes of WT and *SlGRAS7*-OE plants

Parameters	WT	L1	L2	L3
One month Plant height (cm)	16.4±0.36	5.6±1.5**	6.1±1.4**	6.25±1.9**
Two months Plant height (cm)	28.4±2.3	11.2±2.2**	12.4±2.1**	11.6±1.7**
Three months Plant height (cm)	31.2±1.8	18.9±2.1**	19.3±2.5**	19.7±2.6**
One month Primary Root length (cm)	29.2 ± 2.9	14.2 ± 2.2**	13.5 ± 1.9**	15.1 ± 2.3**
Two months Primary Root length (cm)	43.5 ± 3.7	26.3 ± 2.3**	26.9 ± 2.2**	25.3 ± 2.5**
Two months Stem length of sixth internode (cm)	2.4±0.3	0.9±0.2**	1.1±0.2**	1.2±0.3**
Two months Stem diameter of sixth internode (cm)	0.8±0.03	0.5±0.04*	0.5±0.05*	0.4±0.04*
Two months Leave length of sixth node (cm)	5.6±0.05	3.8±0.2*	3.5±0.3*	3.7±0.7*
Two months Leave width of sixth node (cm)	2.3±0.1	1.8±0.3*	1.7±0.2*	1.6±0.1*
Days to first visible flower bud	32.6±2.2	45±2.7**	45.6±2.5**	48±2.7**
Days to anthesis of first flower	47.5±4.0	61.5±2.8**	62.1±2.8**	63.8±2.6**

Note: Values are means of at least more than 30 plants, ± SE. The statistical significance of mean differences was analysed using a *t*-test: *P < 0.05, **P < 0.01.

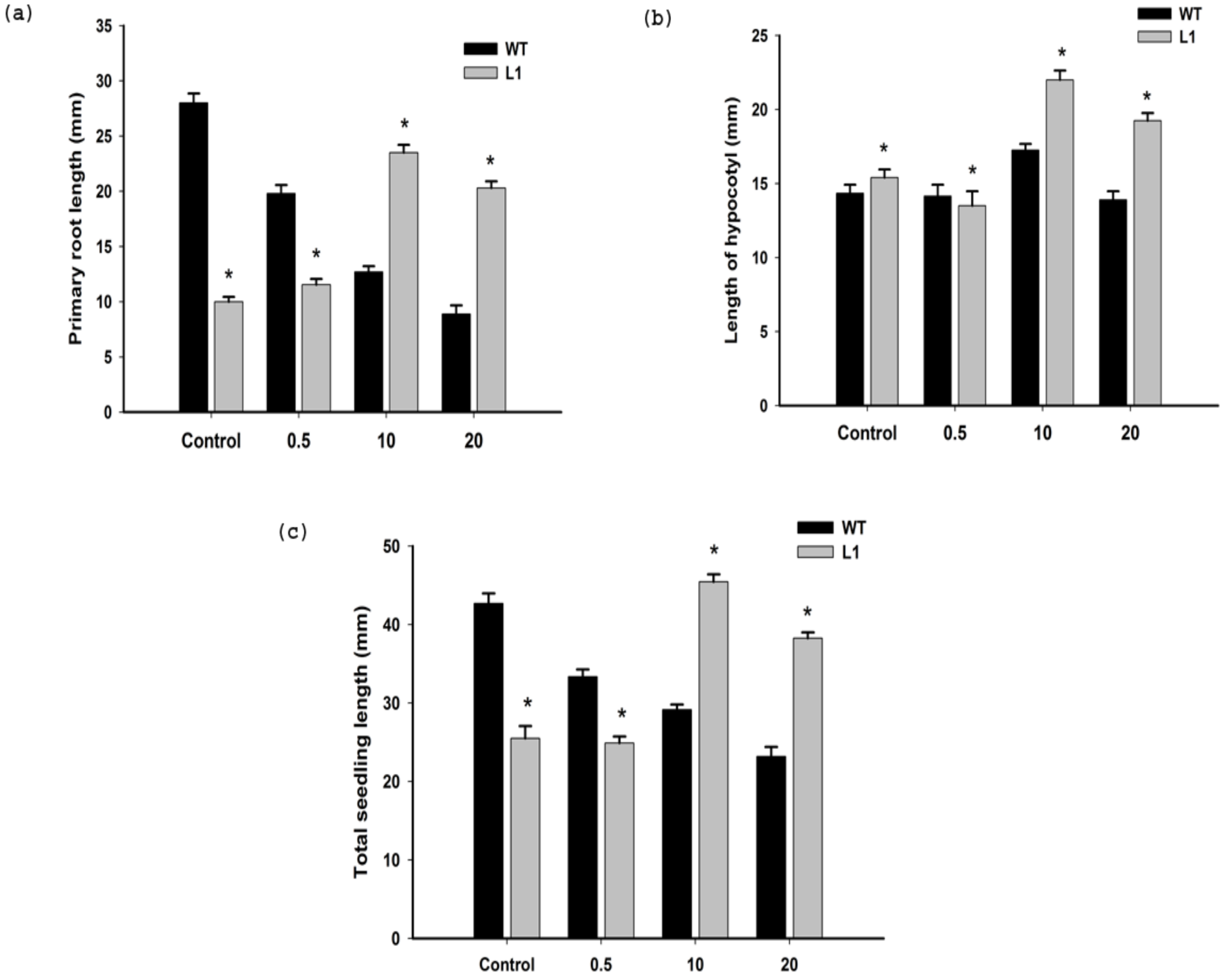

Figure S1. 15 days of WT and *SlGRAS7*-OE L2 seedlings grown on MS/2 medium containing (0µM, 0.5 µM, 10 µM, 20 µM GA_3) (**a**) Primary root length of WT and *SlGRAS7*-OE (L1) seedlings **(b)** Hypocotyl length of WT and *SlGRAS7*-OE (L1) seedlings **(c)** Plant height of WT and *SlGRAS7*-OE seedlings (L1)**.** Asterisks show the significant differences using Student's *t*-test (**P*<0.05, ***P*<0.01).
